# Catering to the patient – development, validation and psychometric properties of an innovative assessment instrument

**DOI:** 10.3205/zma001465

**Published:** 2021-03-15

**Authors:** Miriam Urff, Antonio Krüger, Steffen Ruchholtz, Eva Christina Stibane

**Affiliations:** 1 Universitätsklinik Freiburg, Klinik für Psychiatrie, Psychotherapie und Psychosomatik im Kindes- und Jugendalter, Freiburg, Germany; 2 Asklepios Klinik Lich, Unfallchirurgie, Orthopädie, Wirbelsäulen- und Kindertraumatologie, Lich, Germany; 3 UKGM Standort Marburg, Zentrum für Orthopädie und Unfallchirurgie, Marburg, Germany; 4 Philipps-Universität Marburg, Zentrum für Medizinische Lehre, Marburg, Germany

**Keywords:** assessment, communication, empathy, communication curriculum, medical education

## Abstract

**Introduction:** It has been shown that communication skills acquired during undergraduate medical education are of great importance. Hence, many countries require teaching communication as part of their medical curricula. To assess students’ learning progress, “Catering to the Patient”, as an aspect of showing empathy, should be evaluated. Since there was no description of a validated instrument fitting for this purpose, one had to be developed. To describe its process of development and its psychometric properties were the aims of this study.

**Methods: **Based on the Calgary-Cambridge Observation Guide (CCOG), items describing catering to the patient were selected and modified. Cognitive pretest interviews were conducted to check understandability. Therefore, 7 raters assessed 1 video each (R=7, V=1). In a following pilot study (R=3, V=10) first psychometric properties were evaluated and necessary corrections in the preliminary evaluation form were carried out before the final evaluation form was used to assess students’ ability to cater to the patient and psychometric properties were described in detail (R=2, V=35).

**Results: **The final assessment instrument, “catering to the patient – Marburg evaluation form”, contains 11 checklist items and two global ratings (items 12 and 13). In the final evaluation the inter-rater reliability (IRR) ranged from 0 to 0.562, the median was *r*=0.305. Concerning item 13 (a global rating), 88.6% of the videos were scored with the maximum difference of one point. The internal consistency was very high (Cronbach’s α: α=0.937 and α=0.962), and the correlation between the checklist items and the global rating was high (Pearson’s *r*: *r=0.856 and r*=0.898).

**Discussion: **The assessment instrument “catering to the patient” is suitable for giving feedback and for using it in formative examinations. Its use for summative examinations can be considered. Further examinations should evaluate if a three-point Likert scale could reach higher values and if item 13 can be used as a stand-alone item.

## Zusammenfassung

**Einleitung: **Es konnte gezeigt werden, dass kommunikative Fähigkeiten, die während des Medizinstudiums erworben werden, von großer Wichtigkeit sind. Daher wird in vielen Ländern Kommunikation, als Teil des Curriculums des Medizinstudiums, zu lehren gefordert.

Um den Lernfortschritt von Studierenden zu beurteilen, sollte das „Eingehen auf den Patienten“ als ein Aspekt von Empathie evaluiert werden. Da es keine Beschreibung eines validierten Instruments gab, das für diesen Zweck genutzt werden konnte, musste ein solches entwickelt werden. Das Ziel dieser Abhandlung ist, den Entwicklungsprozess des Instruments und seine psychometrischen Eigenschaften zu beschreiben.

**Methoden:** Basierend auf dem Calgary-Cambridge Observation Guide (CCOG), wurden Items, die das Eingehen auf den Patienten beschreiben, ausgewählt und modifiziert. Kognitive Pretest-Interviews wurden durchgeführt um die Verständlichkeit zu überprüfen. Dafür bewerteten 7 Rater jeweils 1 Video (R=7, V=1). In einer darauffolgenden Pilotstudie (R=3, V=10) wurden das erste Mal die psychometrischen Eigenschaften evaluiert und notwendige Verbesserungen am vorläufigen Bewertungsbogen durchgeführt, bevor der finale Bewertungsbogen genutzt wurde, um die Fähigkeiten von Studierenden bezüglich des Eingehens auf den Patienten zu bewerten und die psychometrischen Eigenschaften detailliert zu beschreiben (R=2, V=35).

**Ergebnisse: **Der finale Bewertungsbogen „Eingehen auf den Patienten – Marburger Bewertungsbogen“ beinhaltet 11 Checklisten-Items und zwei Globalbewertungen (Items 12 und 13). In der abschließenden Evaluation wieß die Interrater-Reliabilität (IRR) Werte zwischen 0 und 0,562 auf, der Median lag bei *r*=0,305. Bezüglich Item 13 (eine Globalbewertung) wurden 88,6% der Videos mit maximal einem Punkt Differenz bewertet. Die Interne Konsistenz war sehr hoch (Cronbach’s α: α=0,937 und α=0,962); die Korrelation zwischen den Checklist-Items und der Globalbewertung war hoch (Pearson’s *r*: *r*=0,856 und *r*=0,898).

**Diskussion:** Der Bewertungsbogen „Eingehen auf den Patienten“ ist geeignet Feedback zu geben sowie zur Nutzung in formativen Prüfungen. Der Einsatz in summativen Prüfungen kann erwogen werden. Weitere Untersuchungen sollten evaluieren, ob mit einer dreistufigen Likert-Skala höhere Kennwerte erreicht werden könnten und ob Item 13 als alleiniges Item genutzt werden könnte.

## 1. Introduction

It is estimated that doctors conduct 200,000 conversations with patients throughout their careers [1]. Just by conducting a thorough medical history, 70% of all diseases can be diagnosed correctly, and with the aid of a physical examination, 90% [2]. These facts alone show the great importance of communication in medicine.

Additionally, the change in the physician–patient relationship in the second half of the 20th century, from paternalism to a patient centered approach, has brought a change to the expectations of physicians’ communication [3].

Physicians can improve their communication abilities. When physicians participated in a one month [4] or 2.5 day module, their communications skills improved [5]. These abilities can already be acquired throughout undergraduate education [6]. It has been shown that the communication skills acquired during undergraduate education are of great importance [7]. Hence, many countries require teaching communication as part of their medical curricula. For example, in Canada, it is integrated into The CanMEDS Physician Competency Framework [8] and in Germany in the licensing act for physicians (“Approbationsordnung für Ärzte”) [https://www.gesetze-im-internet.de/_appro_2002/].

Often, assessment instruments are used for teaching and evaluating students’ communication skills. There are many instruments to rate these skills [9], [10], [11], [12]. Most of them focus on the different steps in a conversation. Norfolk et al. view empathy as a key aspect for developing rapport [13]. Therefore, it is a core competence of doctor–patient communication. Additionally it is known that empathy decreases during medical school and postgraduate medical education, which makes it very important to enhance it in medical education [14]. 

In the context of teaching students communication skills and empathy the responsibles for the communication curriculum of the skills-lab in Marburg discussed, that it would be valuable to evaluate and ensueing teaching students' abilities in catering to the patient. This is not the same as empathy but shows a great overlap. Catering to the patient puts the focus on the activities and behavior while empathy is a basic attitude. 

The Calgary-Cambridge Observation Guide (CCOG) is an assessment instrument developed by S. Kurtz and J. Silverman to teach and evaluate students’ communicational abilities. It comprises in the long version 73 items in seven categories: initiating the session, gathering information, providing structure, building relationship, explanation and planning, closing the session, options in explanation and planning. The short version is structured correspondingly. The CCOG is a well described [1], [15], [16] and widely used instrument [17], [18], [19], [20] that addresses many aspects of doctor–patient communication, including but not only aspects of catering to the patient. Since the assessment instrument “catering to the patient” should be easy to use and focus on aspects of catering to the patient the new evaluation form was developed based on the CCOG.

Since a suitable assessment instrument for evaluating these aspect of catering to the patient could not be found and even Julie M. Schirmer, author of “Assessing Communication Competence: A Review of Current Tools” [10] did not know about a fitting instrument, one had to be developed. 

The aim of this study was to develop an assessment instrument that describes the aspects of catering to the patient as a core competence of doctor-patient communication. It should be easy and time efficient to use and contain good psychometric properties. Therefore, the newly developed instrument should be examined concerning these properties.

## 2. Methods

The assessment instrument “catering to the patient” was constructed and reviewed in a German version. On purpose of publication the instrument was translated into English.

**Figure 1:**
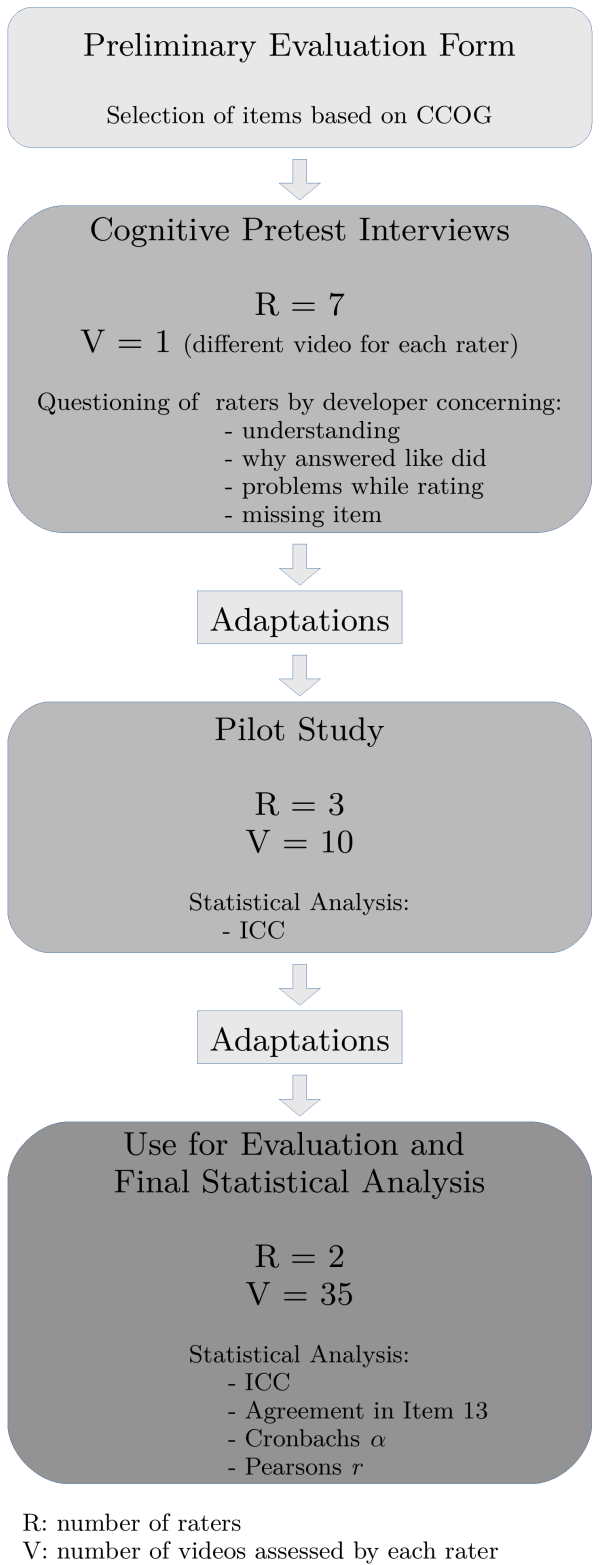
Steps of developing and evaluating the new assessment instrument “catering to the patient”

As it can be seen in figure 1 (Fig. 1) based on the CCOG a preliminary evaluation form was assembled. Following cognitive pretest interviews were conducted. For that purpose seven raters watched each one different video of a doctor-patient encounter (R=7, V=1), in the following they were asked about the wording and idea of the items. Consecutive necessary adaptations were carried out and in a next step it was tested in a pilot study: three raters rated each ten same videos (R=3, V=10). Again, adaptations were made before it was used to assess students’ ability in catering to the patient. Therefore all 35 videos of patient-centered doctor’s advices were assessed by two raters (R=2, V=35). The obtained data were applied to describe its psychometric properties in detail. For each phase different raters were used.

The ethics committee of Philipps-University Marburg approved the study (Az:22/13), and all participants gave informed consent.

### 2.1. Assembling of the preliminary evaluation form

The assembling of the assessment instrument as well as the decisions on how to transform items were mainly conducted by an experienced peer-teacher, a skills-lab leader and an expert of development of questionnaires. Doubtful decisions were discussed with the skills-lab team responsible for the communicational curriculum.

Based on the long version of the CCOG [15], consisting of 73 items, all items concerning the doctor–patient relationship and showing empathy were selected, resulting in 24 selected items (items 6, 10, 12 ,14, 15, 17, 18, 23 - 32, 42, 43, 45, 46, 64, 66, 71). Then, all items that describe a behavior that is not essential for catering to the patient were excluded (items 6, 17, 18, 24, 25, 26, 28, 30, 31, 46, 64). This was done to not rate students worse, who could not show these behaviors due to the observed situation. Some items concerning similar activities were combined (items 12 and 45, 14 and 42). Item 24 was excluded because students in observed situations during the lesson rarely take notes or use a computer. Item 45 had to be split and modified to avoid a double-barreled item. Some items were selected after minor changes (items 10, 23, 29, 32, 42, 66, 71) without changing their meaning; for example in item 23 the explanatory notes were put in brackets. The items then were modified and worded for an observation instrument to fit the format: The student shows a described behavior. To finalize the questionnaire, a global rating (*“****Altogether**** the student has catered very well to the patient’s needs.”*) was added.

The preliminary evaluation form consisted of 11 checklist items and was concluded by a global rating out of rater perspective.

Concerning the scoring, it was decided to use a Likert scale, and additionally short descriptions for each point (“fully applies” to “does not apply at all”). Researching the literature regarding the number of points used when scoring the CCOG showed that five-point Likert scales [12], [21] and three-point Likert scales [1], [22] were used. A five-point Likert scale was chosen to better distinguish between different levels of catering to the patient.

#### 2.2. Cognitive pretest interviews

Qualitative cognitive pretest interviews were carried out to identify any problems concerning the wording of the items and answering categories [23]. Seven students, working as peer teachers in the skills lab, were asked to watch and consecutively rate each one different videotaped interview of a student and a simulated patient (SP). Afterwards, they were asked whether they understood the items, why they chose to answer as they did, whether they had problems understanding any of the items, and whether they felt an aspect of catering to the patient was missing. It was also tested whether a category “not applicable” should be given. For reasons of practicability, pre-existing videos were used, although it was not possible to answer two items: these two items concerned examining the patient and concluding the interview, the interview was stopped after eight minutes and did not include a physical examination. Nevertheless, the videos were suitable because the themes of the discussion were taboo subjects (partnership and sexuality), prompting catering to the patient and developing rapport to a high degree. The topic of the doctor-patient encounter was a patient presenting with worsening of the symptoms of an autoimmune disease. The doctor can learn from the patient that he is not taking his prescribed cortisone because of the side effects and their consequences on his life and his feelings. Initially the videos were recorded for a study concerning difficult topics in doctor-patient communication.

#### 2.3. Pilot study

The same 10 videos that were used before were now watched by 3 different raters using the assessment tool. Two raters were students who work in the skills-lab, and the third rater was an expert who developed the assessment instrument.

Intra class correlation (ICC) was used to assess inter-rater reliability (IRR) because it can be used to compare the ratings of three or more raters. r as coefficient for ICC is confined to an interval from -1 to +1. Since the values of the measurements of reliability are confined to the interval from 0 to +1, negative values show a reliability of 0 [24]. Values of 0.25<*r*<0.5 describe a moderate correlation, and *r*>0.5 describes a strong correlation [25]. All items with a reliability of *r*<0.25 were modified after the pilot study.

#### 2.4. Use of the assessment instrument and final statistical analysis

The last step was to use the evaluation form to asses students’ ability in catering to the patient and to evaluate the assessment instrument on a larger sample. Therefore, two raters rated 35 videos. One rater was an anesthesiology resident, and the other was a resident in child and adolescent psychiatry and psychotherapy. The videos were recorded in connection with a study that evaluated the effectiveness of a course for medical students about difficult doctor–patient encounters.

Each of the videos showed the interaction between a student and an SP. The students’ task was each time to discover the reason for the patient’s admission to the hospital (including taking a patient’s history and physical examination); and informing the patient about follow-up procedures. The setting for the appointment was a patient with symptoms suggesting a bronchial carcinoma. He would like to discuss his fear of pending death with the doctor. The underlying educational topic of discussion is catering to the patient. The duration of the videos is about 20 minutes.

IRR was assessed again, to detect whether the changes in the assessment instrument made after the pilot study were effective. For comparability, ICC was used again. Additionally it was compared how often the residents gave the same rating for item 13. The percentage of agreement as well as the percentage of difference of one, two, three or four points were analyzed. Internal consistency was evaluated using Cronbach’s α [26]. α may vary between -∞ and +1; ideally it reaches values around 0.9 [27]. To assess whether the global rating of item 13 reflects items 1 to 11, an index was created. This index was determined for each video by calculating the mean value of items 1 to 11. Then the correlation between the index and item 13 was assessed. (For calculating the index, item 12 was not integrated because it is a global rating from the patient’s perspective).

## 3. Results

**Figure 2:**
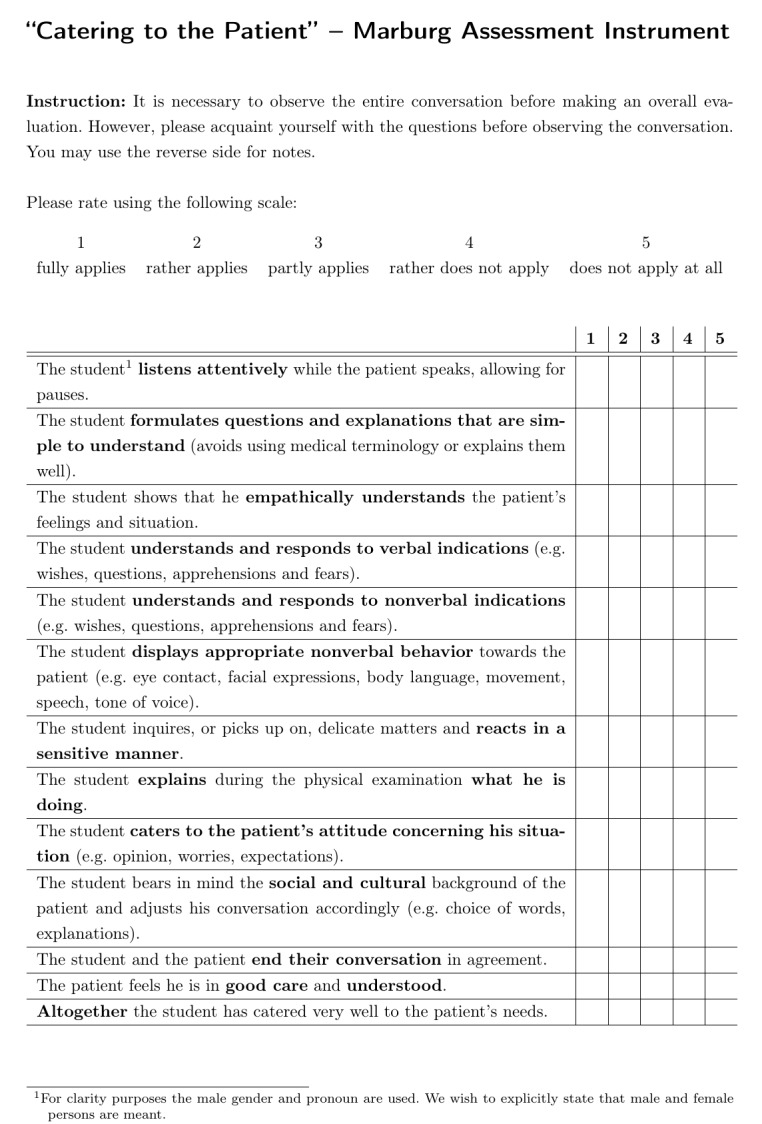
Assessment instrument “Catering to the Patient”

The results of the cognitive pretest interviews, the following adaptations, the pilot study, and the subsequent changes in two items led to the assessment instrument “catering to the patient – Marburg evaluation form” shown in figure 2 (Fig. 2). Its psychometric properties were evaluated in a final statistical analysis.

### 3.1. Results of the cognitive pretest interviews

As a result of the pretest interviews, some minor changes in the wording of the existing items were made. Furthermore, an additional item was integrated, regarding a global rating from the patient’s perspective. It was included as item 12, after the items addressing different behaviors and before the global rating from the observer’s perspective.

The pretest showed that if the answering category “not applicable” was given, items that could be judged often were not answered. Therefore, it was decided to not include that option.

#### 3.2. Results of the pilot study

Within the pilot study, ICC was calculated to evaluate IRR. The results are shown in table 1 (Tab. 1).

Items 8 and 11 could not be evaluated because no physical examination took place and the conversation was stopped after eight minutes. Items 5, 6, 12 and 13 showed moderate correlation between raters and items 1, 4, 7, 9 and 10 showed strong correlation. Due to their good reliability, these items were not changed. Items 2 and 3 showed a reliability of 0, therefore changes had to be made within the items. It was observed that these two items were double-barreled, which might have caused their low reliability.

Hence, item 2, which originally read: “The student formulates questions and explanations that are exact and simple to understand (avoids using medical terminology or explains them well)” was changed to “The student formulates questions and explanations that are simple to understand (avoids using medical terminology or explains them well)”. The wording of the item was changed to assess only whether the questions were simply formulated. In doing so, the patient’s understanding is taken into consideration.

Item 3 also had to be modified. The item originally read: “The student shows that he emphatically understands the patient’s feelings and situation (e.g., by verbalizing, paraphrasing)” was changed to “The student shows that he emphatically understands the patient’s feelings and situation”. It was referred that the examples complicated the rating, therefore they were excluded.

#### 3.3. Description of the assessment instrument, “catering to the patient”

The assessment instrument used for the final assessment is shown in figure 2 (Fig. 2). It contains 13 items, of which 11 describe different behaviors and two are global ratings from the patient’s as well as the observer’s perspective. They are rated with the aid of a five-point Likert scale, labeled with 1=fully applies, 2=rather applies, 3=partly applies, 4= rather does not apply and 5=does not apply at all.

The 11 checklist items contain

1. listening attentively,
2. formulating questions and explanations that are simple to understand,
3. showing emphatically understanding,
4. understanding and responding to verbal indications,
5. understanding and responding to nonverbal indications,
6. displaying appropriate nonverbal behavior,
7. reacting in a sensitive manner,
8. explaining during the physical examination what one is doing,
9. catering to the patient’s attitude concerning his situation,
10. bearing in mind the social and cultural background, and
11. ending the conversation in agreement.

The two global ratings are out of patient’s perspective (The patient feels he is in good care and understood) and out of rater’s perspective (Altogether the student has catered very well to the patient’s needs).

#### 3.4. Final statistical analysis

The analysis of the psychometric properties in the final assessment included an evaluation of the IRR, the agreement between the raters in item 13, internal consistency, as well as the correlation of the index with item 13.

The results of the ICC (see table 2 (Tab. 2)), expressing the IRR, range from *r*=-0.153 to *r*=0.562. The median is *r*=0.305. It can be seen that the reliability of items 2, 6 and 10 is high. Items 1, 4, 5, 7, 11 and 13 show moderate reliability. Item 3 shows low reliability and items 8, 9 and 12 show a reliability of *r*=0.

Table 3 (Tab. 3) shows the results of agreement in item 13. All videos scored with a 1 by rater 1 are displayed in row 1. The columns indicate how rater 2 rated these videos. So five videos were scored with a 1 by both raters; two videos were scored with a 1 by rater 2 and a 2 by rater 1. It can be seen that 45.7% (n=16) of the videos were scored the same by both raters and there is only a one point difference in the ratings for 42.9% (n=15) of them. Those that differ by two points constitute 8.6% (n=2), and those with a three-point difference, 2.9% (n=1).

Internal consistency of the assessment instrument is evaluated by using Conbach’s a. With α=0.937 for rater 1 and α=0.962 for rater 2 it shows excellent values and therefore a high interrelatedness and internal consistency of the items.

Table 4 (Tab. 4) shows the means of the index and item 13 as well as Pearson’s r to describe the correlation. The correlation between the index and item 13 for rater 1, using Pearson’s *r*, is *r*=0.856, and for rater 2, *r*=0.898. The significance for both values is *p*<0.001. This indicates a high correlation between items 1 to 11 and item 13.

## 4. Discussion

The CCOG is a widely used instrument for teaching [18] and measuring [17], [19], [20] communication. The short version of the CCOG was translated into German and examined by a German research group. The test-retest reliability and validity are good, therefore it is suitable for evaluating medical students’ communication abilities [16].

The purpose of this study was to develop an assessment instrument to measure students’ ability to cater to the patient. The instrument should be easy to use and time-efficient. Furthermore, its psychometric properties should be good.

### 4.1. Choosing the answering category

Simmenroth-Nayda et al. used a three-point Likert scale in their pretest, but changed it to a five-point Likert scale because the pretest revealed problems with ratings, which led to that change [16]. On the other hand, a three-point scale might have been able to reach higher values for IRR [28]. In the future, it needs to be decided whether a higher IRR is necessary, in which case a three-point scale could be used. Otherwise, if a more differentiated rating is important (e.g., for giving feedback), then a five-point scale should be used. As described in 2.1, it was decided to use a five-point Likert scale.

#### 4.2. Statistical analysis

The statistical analysis suggests that the assessment instrument is feasible for teaching and formative examinations. For summative examinations it can be recommended, keeping in mind that some of the results are only moderate.

The results of the pilot study showed that the reliability of items 2 and 3 is 0. When the ICC was calculated in the final assessment, these items showed an ICC-coefficient of *r*=0.562 for item 2 and *r*=0.205 for item 3. These results suggest that the changes were, especially for item 2, effective. Both calculations of IRR showed moderate values for the medians: in the pilot study, the median was *m*=0.471, and in the final assessment, *m*=0.305. It is possible that a three-point Likert scale might have led to higher values [28]. A German evaluation of the short version of the CCOG observed similar results to the ones discussed in this paper [16]. They reported IRR-coefficients between 0.05 and 0.57.

Another possibility to improve the reliability might be through rater training [29]. Although rater training varies, and in some cases the raters only receive information via e-mail [30], it was decided not to conduct any rater training. The decision was made because training, even in a digital way, is time consuming, which conflicts with the aim of a simple and time-efficient assessment instrument. Additionally, often no rater training occurs when instruments, such as this one, are used outside the context of a study. Therefore, the antecedent study must also be done without any rater training.

Concerning item 13, 45.7% of the videos were scored the same by both raters, while 42.9% of the videos were scored with a one-point difference. This results in 88.6% of the videos being scored with no more than a one-point difference. Whether this is acceptable depends on the purpose of its usage. For formative examinations and feedback it is suitable. Before using in a summative evaluation, one should consider whether the score should be used alone or combined with scores of other assessment instruments.

For rating complex skills like communication, global ratings are better suited than checklists [30], [31]. In this evaluation it could be shown that the correlation between the checklist items (items 1 to 11) and the global rating (item 13) is very high (*r*_1_=0.856, *r*_2_=0.898). Additionally, excellent internal consistency was reached (α_1_=0.937, α_2_=0.926). Based on this data it could be deduced that item 13 by itself is sufficient to rate the catering to the patient. On the other hand, the checklist items probably teach the rater about the criteria for evaluation before the global rating is given. Thus, whether item 13 by itself is suitable for summative examination needs further evaluation. For formative examination the items can be used to give more detailed feedback.

#### 4.3. Critical limitations

Focusing on the critical limitations first the small samples are to be pointed out. Already during the pilot study a greater amount of data would have helped to improve the items. Especially it would have been advisable to conduct a second pilot study to figure out if the changes made were effective. In the final statistical analysis it would have been advantageous to integrate the data of a larger number of raters to get better values for IRR. If the number of videos had been higher, statistical analysis would have been sounder.

Another limitation is the aim to measure the construct of “Catering to the Patient”. The decision to assess this concept arose from practical needs, although it is seen as good scientific practice to select the aim of measuring on the basis of theoretical considerations or literature research. Additionally there was no conceptual preliminary study, so the construct is not well defined. Therefore, a clear differentiation between empathy, developing rapport and catering to the patient was not made. Otherwise, it is an opportunity to use a tool that is based on practical needs.

In the study out of practical reasons only videos showing conversations between students and SPs were rated. It might be that the observation of direct encounters as well as conversations between real patients and students would show different results. It is reckoned that the differences between SPs and real patients is small if SPs act well. The difference between videotaped encounters and live encounters is hard to estimate since there might be differences in what the rater can observe e.g. the gestures and facial expression as well as the atmosphere between the two parties. In this study the raters were not allowed to re-watch scenes or videos because this would probably make a big difference compared to live encounters. Additionally when in use outside of a study there would probably not be enough time to watch videos or even parts of them several times.

The aim was to establish an assessment instrument that is simple to use. Nevertheless it is necessary to take some minutes before observing the encounter to get acquainted with the items. Since it is fundamental to watch the entire conversation before rating, again time is needed after the encounter to conduct the rating. (That is why) To make it as time-efficient as possible, the data arising from this study were produced without any rater trainin

Another limitation is about the language. In this study the validation was conducted using the German version. Only for this article it was translated into English. Additionally it is suggested to take different steps in the translation process e.g. a back translation and various reviews [32]. In this case the evaluation form was translated by the author and a native speaker in English, both with excellent knowledge in the other language, without any further translation process.

#### 4.4. Future prospects

Further studies could analyze if the patient’s or SP’s evaluation of the students’ ability to cater to the patient correlates with the assessment of a not involved observer. For this item 12 (global rating out of patient’s perspective) can be used.

This assessment instrument has a wide range for future use. As described above it can be deployed in training as well as in examination, bearing in mind that its use in summative evaluations has to be critically checked. In this study it was only utilized to evaluate videotaped situations but it is imaginable to be used in live encounters as well. It is estimated that it can be applied in all levels of the medical education. It was tested in two undergraduate settings (pilot study and final statistical analysis) but the use in a postgraduate setting is conceivable as well.

Probably most people think about employing the instrument in subjects focusing on the conversation with the patient but it can be used in other subjects as well. The topics of the encounter recorded in the videos of the final statistical analysis were in connection with teaching surgical students. Even though it could be used in all kind of situations it will probably be most advantageous in training or assessing of demanding situations.

## 5. Conclusion

The psychometric properties of the assessment instrument “Catering to the Patient – Marburg Evaluation Form” suggest that it definitely can be used for teaching and formative examination. Its use can be considered for summative examination. Nevertheless, it should be evaluated whether a three-point Likert scale could reach higher values of IRR. Furthermore, it would be interesting to examine whether item 13 as a stand-alone item could be used, since this was considered to be controversial.

## Acknowledgements

We thank all the students and raters who contributed to the success of this study. Particularly we want to thank Lorianna Könneker for her help in translating the assessment instrument. We also want to acknowledge the financial support of the Department of General Medicine of Philipps-University Marburg, who made it possible for the raters to receive an expense allowance; the “Förderverein Maris e.V,” who paid for the costs of the proofreading service; and the skills-lab “Maris” itself for covering the expenditure on the peer teachers’ wages.

## Profiles

**Name of school:** Philipps University Marburg

**Study program/occupation: **Medicine

**Number of students per year and/or per semester:** 350 p.a.

**Has a longitudinal curriculum covering communication been implemented? **Yes

**At which semester levels are communicative and social competencies taught?** 1, 2, 5, 6, 7, 8, 9, (PJ)

**Which teaching formats are used? **Courses, practical courses, simulation with simulated patients

**During which semesters are communicative and social competencies tested (formative, pass/fail, graded)? **Formativ: 2 and 9, summative with marks 5 and 6

**Which assessment formats are used?** Roleplay, video feedback, OSCE with simulated patients

**Who (e.g. hospital, institution) is in charge of development and implementation? **Interdisciplinary skills lab in cooperation with clinical departments

## Current professional roles of the authors

- Dr. med. Miriam Urff studied medicine in Marburg and is assistant doctor at the University Medical Center Freiburg, Clinic for Psychiatry, Psychotherapy and Psychosomatics in Childhood and Adolescence. Three years she was student tutor in the Marburg interdisciplinary skills lab and conducted a project for doctor-patient´s communication in surgery for her dissertation.
- Prof. Dr. med. Antonio Krüger is the clinic's head physician for trauma surgery, orthopedics, spine and pediatric trauma at the Asklepios Clinic in Lich. He was a senior physician at the university clinic in Marburg for many years, and he was lecturer and agent for students in the practical year. He is convinced that teaching interdisciplinary - for example in skills labs - can improve learning.
- Prof. Dr. med. Steffen Ruchholtz is Director of the Center for Orthopedics and Trauma Surgery at the university hospital Marburg. As a representative for the Trauma Network Committee of the German Society for Trauma Surgery he is also responsible for the implementation of a concept to optimize the clinical quality of treatment for seriously injured patients in Germany.
- Dr. phil. Tina Stibane heads the Center for Medical Teaching and Learning at the Philipps University of Marburg. She coordinates the skills lab Maris, supervises various teaching-learning projects and is active part of the curriculum committee. She also conducts the medical-didactic training for lecturers and tutors in the medical department.

## Data

Data for this article are available from the Dryad Digital Repository: https://www.doi.org/10.5061/dryad.qjq2bvqcw [33]

## Competing interests

The authors declare that they have no competing interests. 

## Figures and Tables

**Table 1:**
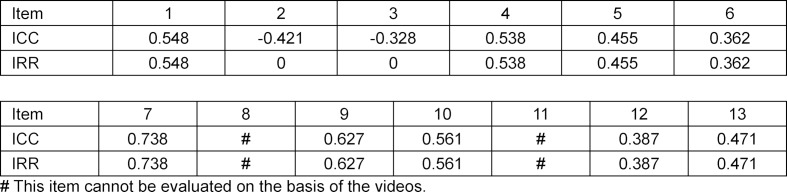
Intra class correlation (ICC) and following inter-rater reliability (IRR) of pilot study

**Table 2:**
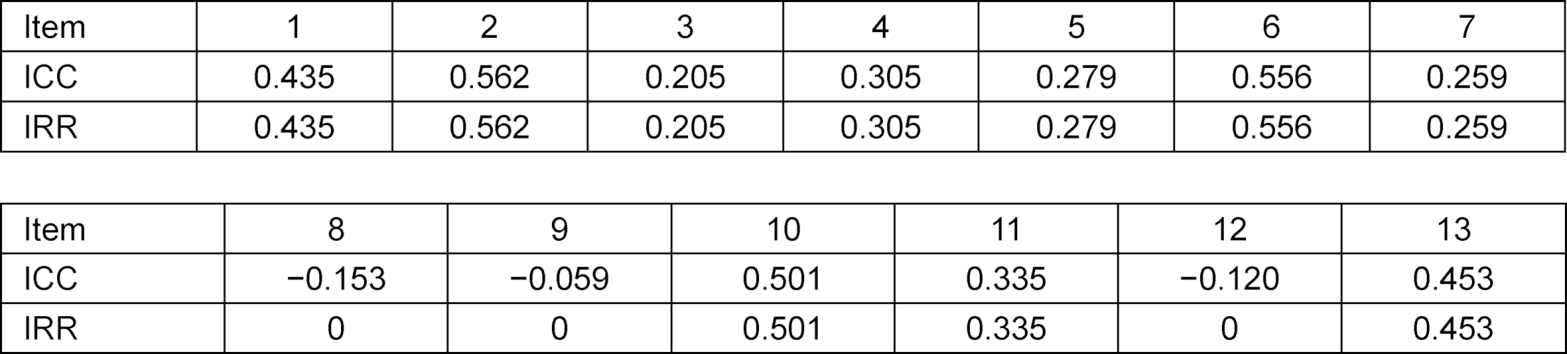
Intra class correlation (ICC) and following inter-rater reliability (IRR) of final assessment

**Table 3:**
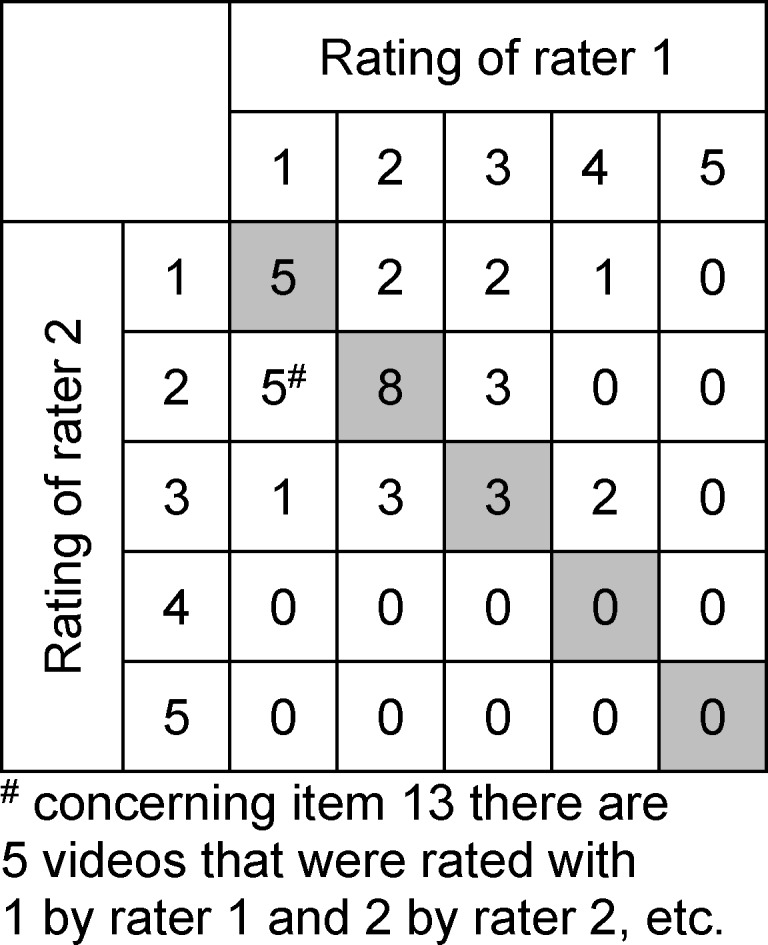
Agreement in item 13 between raters

**Table 4:**
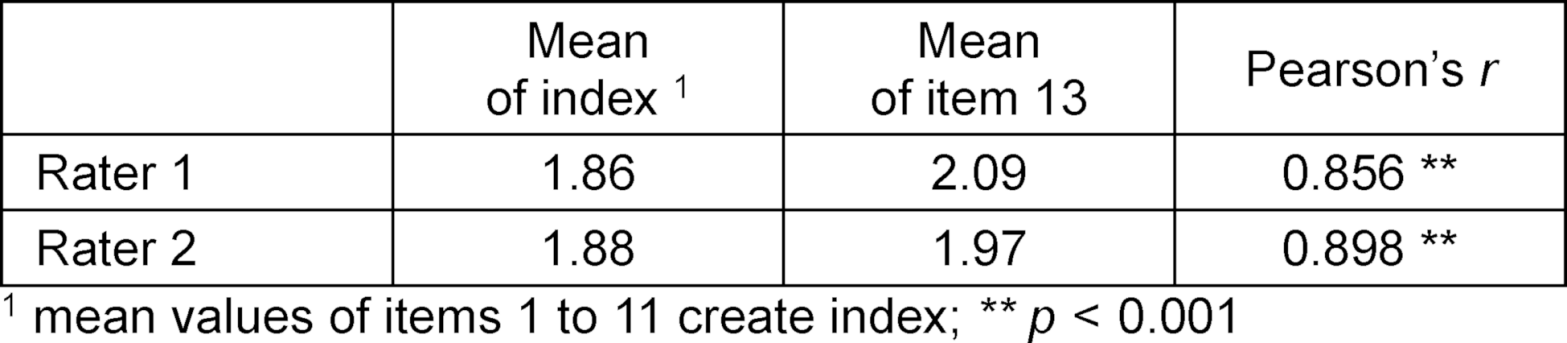
Correlation of index and item 13
